# Follow-up in patients treated for head and neck cancer

**DOI:** 10.1007/s12254-014-0143-y

**Published:** 2014-05-21

**Authors:** Andrzej Kawecki, Romuald Krajewski

**Affiliations:** Head and Neck Cancer Department, Cancer Center—M. Sklodowska–Curie Memorial Institute, ul. Roentgena 5, 02-781 Warsaw, Poland

**Keywords:** Oncological follow-up, Head and neck cancer, Recurrence, Metastases, Second primary tumor

## Abstract

Principles of follow-up management in patients treated for head and neck tumors are not very strictly defined, and practice varies between countries, centers, and specialists. Details of follow-up procedures, including timing of outpatient visits and diagnostic imaging, are specific for tumor types and localizations but also depend on treatment modalities used, availability of diagnostic procedures, and socioeconomic factors. The authors describe general principles of follow-up in head and neck cancer patients. Clinical observation and laboratory and imaging studies in patients who had received radical treatment should focus on early identification of recurrent or second primary tumors to allow for a salvage radical therapy. In patients who initially had received a palliative care, the follow-up should focus on proper management of symptoms.

## Introduction

Management of head and neck cancer (HNC) is a serious challenge worldwide. In 2012, ~ 700,000 persons have been diagnosed and more than 370,000 died of HNC [1]. Standard radical therapy in early-stage HNC is surgery and/or radiotherapy (RT). Up to two-third of patients present with an advanced HNC, and surgical resection with adjuvant RT or radiochemotherapy (RTCT), RTCT alone, or RT with cetuximab are used. Overall prognosis is still unsatisfactory.

During 5-year follow-up, locoregional recurrence occurs in 25–50 % of patients with advanced cancer [2, 3]. Second primary cancers occur in 3–5 % of cases per year [4]. Salvage surgery, RT retreatment, chemotherapy (CT), and combined radical treatment can be implemented more frequently, and results are better if an early diagnosis of recurrence or of new cancer is made; however, it is possible in only up to 20 % of patients, and only 5 % with recurrence survive 5 years or more. In the majority of cases, only a palliative CT or supportive care is indicated [2, 5, 6].

Another goal of follow-up care is detection and management of late complications that occur in 10–80 % of patients who underwent treatment for HNC [7–10]. Comorbidities are frequent in this group and require coordinated management. Dental care; psychological, emotional, and social support; and elimination of exposure to risk factors that contributed to primary cancer are also important. Proper care for all those aspects by an interdisciplinary team has been shown to prolong survival and its quality [10, 11].

It is advisable to continue follow-up by the team that was involved in oncological management of the patient, with help from other specialists and professionals caring for oncological patients [12]. Professionals not familiar with problems of such patients might be reluctant to care for them, and it may result in a suboptimal care or in unnecessary procedures.

There is no commonly accepted standard of follow-up care in patients treated for HNC, and evidence supporting every aspect of this care is weak. Efficacy and cost-effectiveness of different schedules, laboratory tests, and diagnostic imaging have not been established. Reviews show very large variability of existing practice [13–15]. In this short paper, based on nonsystematic review, we present only the most frequently recommended procedures. Due to complexity of HNC management and lack of strong evidence supporting recommended follow-up procedures, it is necessary to adapt these recommendations to individual situation of each patient.

## Detection of locoregional recurrence, distant metastases, and second primary tumors

### How frequent should be follow-up outpatient visits?

Published guidelines indicate that check-up visits in an oncology outpatient service should take place every 1–2 months during the first 6 months after completion of treatment, every 2–3 months in the next 6 months, every 3–4 months during second year, and every 6 months during years 3–5 [12, 13, 16]. The visit 3 months after treatment is particularly important because at this time, the baseline result of treatment should be established [17]. Besides detailed clinical assessment, a baseline imaging study is also advisable for all patients in whom direct or endoscopic visualization and palpation are not reliable or insufficient. If a complete response or radical resection has not been achieved, available options of radical retreatment have to be considered. If the disease persists and there are no radical treatment options, the follow-up should continue according to principles described later in the text for patients who received a palliative treatment.

For a long time, the 5-year active oncological follow-up had been considered sufficient. However, HNC patients run 3–5 % yearly risk of second primary cancer throughout their life, particularly if exposure to carcinogens continues. After 5 years, follow-up visit is advised every year [16], but effectiveness of this approach is not clear [18].

### Which laboratory tests and diagnostic imaging are indicated?

Each follow-up visit should include careful history taking because presence of symptoms has been shown to indicate higher risk of recurrence [5, 18]. Detailed systemic and site-specific examination is mandatory. With improved availability, an endoscopic examination has become a routine part of clinical assessment. Recorded endoscopic images can facilitate detection of a recurrence or a new tumor.

In localizations poorly accessible or not accessible to direct or endoscopic vision and/or palpation, diagnostic imaging is an indispensable part of follow-up.

### Diagnostic imaging

CT scanning or MRI or both are indicated at the outset of observation. Afterward, these examinations are indicated in symptomatic patients, but should not be advised as a routine. Imaging might be indicated when clinical assessment is not reliable [12, 16].

Ultrasound imaging of the neck is an easily available and inexpensive option in follow-up of regional lymph nodes. When coupled with fine-needle biopsy. it has high sensitivity and specificity, and can be used to improve accuracy of clinical neck examination [13].

Role of diagnostic imaging in detection of systemic spread is not clear. Traditional advice was to perform chest X-rays every year, but its sensitivity is low [13, 17]. In patients with high risk of second primary cancer (usually due to continuing smoking), yearly low-dose CT scan of the chest would be advisable [16]. Imaging of other organs/regions is advised only when indicated by symptoms.

### Functional imaging

Availability and use of positron emission tomography (PET) do increase, and new modalities of imaging are introduced. Usefulness and effectiveness of these methods in routine follow-up are under investigation [19]. At present, PET scanning is not recommended in routine follow-up of patients treated for HNC, mainly due to relatively high rate of nonspecific, falsely positive results. PET is useful as a second-line diagnostic procedure in patients suspected of recurrent or second primary tumor when other diagnostic imaging procedures and biopsy are insufficient to confirm or rule out the cancer [17]. It is also increasingly used in staging of patients who are considered candidates for major salvage surgery procedures and in assessment of treatment results.

### Laboratory tests

In HNC, there are no tumor cell markers that can be monitored during follow-up and used as an early indicator of recurrence [12]. Use of routine laboratory tests should be adapted to the treatment the patient had received and its toxicity. One specific indication is monitoring of thyroid function if the gland was included in irradiated field. Hypothyroidism occurs in up to 40 % of those patients, and TSH assessment every 6–12 months is advised [13, 16]. Approximately 15–30 % of RT/RTCT patients have nutritional problems that require monitoring of liver function, protein level, and other nutritional parameters for as long as these problems persist [16, 20].

### Biopsy

As mentioned earlier in the text, early diagnosis of recurrence has a positive impact on prognosis. Taking this into account, an active approach even to apparently “benign” symptoms and lesions is advisable. During follow-up, a standard initial attempt to histologically verify tissue suspected of persistent/recurrent/new cancer is the fine-needle biopsy. Its accuracy can be enhanced by ultrasonographic or CT guidance. Positive results confirm recurrence, but negative/nondiagnostic results are of a limited value. If the lesion progresses, an incision biopsy should be done. For superficial lesions, a punch or incision biopsy under local anesthesia is recommended to provide definitive histopathological diagnosis.

## Monitoring and management of treatment sequels, toxicity, and complications

Early and late undesirable effects appear after all types of oncological management and affect 10–90 % of patients [10]. Recently, there have been a growing number of patients treated with RTCT who run cumulative risk of complications. Complications and sequels of surgical resection usually occur early in postoperative period and tend to improve with time, while tissue damage due to RT and CT may be progressive and irreversible [21].

### Radiotherapy

Early complications of RT include mainly acute mucosal reaction with dysphagia, pain, and xerostomia. Currently, skin reactions are less frequent due to progress in radiation technology. Intensity of early complications is dependent on fractionation scheme. Addition of CT very significantly increases frequency of early complications, but with exception of xerostomia, they tend to disappear within a few months after treatment.

Severe late complications of radical RT occur in 10–15 % of patients [22]. Detection of late radiation toxicity is based on detailed search for symptoms and signs. The most frequent are xerostomia and mucosal reactions leading to difficulty in swallowing and significant deterioration of life quality [21]. Necrosis of the bone or cartilage occurs in 5–8 % of patients [10]. Tissue necrosis usually requires surgical treatment. Conservative symptomatic treatment and physical methods are indicated to alleviate symptoms.

### Surgery

Radical resection of an early HNC has low frequency of complications. However, majority of surgically treated patients have an advanced cancer and undergo complex, extensive surgical procedures. During early follow-up, particular attention should be paid to signs and symptoms of surgical site infection, as majority of these infections become apparent after discharge from hospital [23]. Early rehabilitation is advised to prevent shoulder, temporomandibular joint, speech, and swallowing dysfunction. Scarring, fibrosis, and contractures require early detection, physiotherapy, rehabilitation, and additional surgical interventions.

Sequels of surgical resection, such as deformities of face, impaired chewing and swallowing, speech disturbances, and stricture of the pharynx require constant attention during follow-up. Initially, oncological aspects take precedence over other concerns, but as risk of recurrence diminishes with time, sequels of therapy become increasingly important. An interdisciplinary team familiar with management of oncological patient is necessary to assure proper management of these complex problems.

### Radiochemotherapy

In HNC, cumulative doses of chemotherapeutics usually are not very high, but combined risk of early and late complications in patients receiving RTCT is significant [10]. Hematologic complications are the most frequent during treatment and occur in 37 % of cases, while the most frequent late complication in this group was dysfunction of pharynx/esophagus in 7 % [24]. Patients with adverse reactions due to CT should undergo regular laboratory tests. Management of CT complications is symptomatic.

Patients with HNC frequently have dysphagia, and it has major impact on quality of life. It is present before treatment in at least 10 % of patients, during treatment and in early follow-up period in 80 % or more, and then gradually improves. Underreporting by patients is common [25–27]. It is important to actively check for dysphagia symptoms using standardized assessment tools. Loss of weight might indicate problems in swallowing. Xerostomia is a frequent contributing factor. Up to several percent of patients after RT/RTCT to the neck develop esophageal stricture that causes severe dysphagia and requires mechanical dilation or other surgical management.

## Follow-up after palliative care

In patients who received only palliative care, the management during follow-up should concentrate on the quality of life. When no new symptoms appear, follow-up visits can be reduced to a single visit 1–2 months after treatment completion. Diagnostic imaging is indicated only to guide palliative symptomatic procedures, particularly management of pain and maintenance of the airway. Follow-up of this group of patients should be coordinated by palliative care team.

### Prevention of second primary tumors

Risk of second primary tumor can be reduced by elimination or reduction of exposure to carcinogens [28]. A consultation and support from specialists in management of dependence should be advised. There are no specific medicines or supplements that have been proven to reduce risk of a new cancer or of the recurrence. Patients should be advised to maintain normal body weight, to pursue physical activity, and to eat regularly with particular attention to fruits, vegetables, and grain foods [29].

It is important to stress that published recommendations on timing of visits and investigations to be carried out are mostly based on expert opinion and observational or retrospective studies and reflect institutional practice. In comparison with NCCN guidelines [16], the recommendations in this paper are more detailed and include some additional procedures, like ultrasonography imaging with fine-needle biopsy in the evaluation of neck lymph nodes. The NCCN guidelines include an advice on dental evaluation in patients treated for oral cavity tumors and after RT involving oral cavity. We included this important part of follow-up care among psychological, emotional, and social support and elimination of exposure to risk factors to indicate the need for a comprehensive, interdisciplinary care. NCCN also included an option to monitor EBV status in nasopharyngeal cancer patients. In our paper, we do not include site-specific advice, but it is stressed that in view of insufficient evidence and large variation in published practices, it is necessary to consider the situation of each patient on an individual basis.

## Summary

Standard follow-up of patients treated for HNC is based on regular follow-up visits, with frequency decreasing in consecutive years. Imaging with CT and/or MRI is indicated to establish treatment results and baseline stage for reference during observations. Ultrasonography of the neck is useful in assessment of regional lymph nodes. The most important goal of observation is an early diagnosis of recurrence or second primary tumors that undertake salvage therapy and improves prognosis. Patients treated for HNC present with wide range of anatomical, functional, and emotional problems for which follow-up management by a multidisciplinary team should be available.

### Take-home message

The main goal of patient observation during follow-up after treatment for a head and neck cancer is an early detection of recurrence or of second primary tumor. Observation and management during follow-up should be carried out by a multidisciplinary oncological team.

### Conflict of interest

The authors declare there is no conflict of interest.

### Open Access

This article is distributed under the terms of the Creative Commons Attribution License which permits any use, distribution, and reproduction in any medium, provided the original author(s) and the source are credited.
